# A Multi-Stakeholder Perspective on the Integration of Safety in University Nursing, Education, and Engineering Curricula

**DOI:** 10.3390/ijerph15071429

**Published:** 2018-07-06

**Authors:** Paul Rothmore, Arthur Saniotis, Dino Pisaniello

**Affiliations:** School of Public Health, Faculty of Health and Medical Sciences, The University of Adelaide, Adelaide, SA 5005, Australia; arthur.saniotis@adelaide.edu.au (A.S.); dino.pisaniello@adelaide.edu.au (D.P.)

**Keywords:** safety, university curricula, education

## Abstract

Improvement in workplace safety is dependent upon the active engagement of workforce leaders and designers. The university sector plays a key role in the education of these future leaders, and there is an expectation that safety education in universities will encompass more than just a safe learning environment—that is the nurturing of broader safety attitudes and awareness. However, with the exception of dedicated safety training programs, safety education is often delivered and assessed on an ad-hoc basis and at academic discretion. This is partly due to the absence of a simple tool with which curricula can be evaluated from a safety perspective. In a qualitative approach, semi-structured interviews were undertaken with multiple stakeholders (academics, professional organizations, and students) to determine their views on existing safety content in university curricula and on the level of preparedness, from a safety perspective, for workforce entry. University participants came from nursing, mechanical engineering, and education schools at three universities. A simple curriculum evaluative tool was also validated. Results indicated there were divergent views on the level of preparedness for workforce entry both between schools and stakeholder groups. However, the limitations of university curricula were acknowledged. The evaluation tool was shown to provide positive feedback on existing, but previously unacknowledged, safety content and also highlighted areas for future improvement and integration. However, voluntary utilization of the tool was a challenge for busy academics.

## 1. Introduction

Improvement in workplace safety is often dependent upon the engagement and involvement of workforce leaders and workplace designers. Their long-term approach and attitude to this issue may be influenced by early exposure to the identification, assessment, and control of workplace hazards as young workers themselves or as part of formal education [1].

In particular, the university sector plays a key role in the preparation of workplace leaders, operating as an agent of social and community reform [2,3]. Among its students will be the future designers, end-users, or influencers of the work environment. Thus, safety content in curricula can be seen to facilitate safe learning in undergraduate programs, but also to influence attitudes of future professionals.

Safety-related learning is often delivered and assessed on an ad-hoc basis within university coursework programs. Rather than be a formally planned aspect of curricula, safety education is a “hidden curriculum” [4]. This is, at least partly, due to the lack of any such requirement by course accreditation bodies [5,6,7]. Furthermore, there appears to be no curriculum appraisal tool with which courses and programs of study can be evaluated from a safety content perspective. Attempts to gather information on the inclusion of safety content in university curricula have met with limited success. According to a recent EU Report [5],

The greatest challenge is to mainstream occupational safety and health (OSH) into university education in order to reach future engineers, architects, medical professionals, business professionals, managers, etc. The mainstreaming of OSH into university-level courses is the least well-developed area for various reasons, which include the lack of direct national government control over university teaching. Actions to include OSH in relevant courses such as engineering or business studies are therefore ad hoc, and often dependent on the interest of individual professors or particular advocates within professional bodies. Professors need convincing of the need to include OSH in courses. They also need relevant materials.

There is a paucity of published research on the integration of safety-related content into the university curriculum, other than for specialist, safety-focused undergraduate and postgraduate courses.

Jackson, de Munk, and Elms [3] considered the extent to which health, safety and environment education was implemented into Master of Business Administration (MBA) courses throughout Australian universities. In response to the finding of a significant absence of safety-related components within MBA courses throughout Australia, and given that the inclusion of safety information is professionally relevant, the authors recommended that relevant content be integrated into existing tertiary coursework.

In 2009, Stacey et al. [7], in conjunction with the UK Health and Safety Executive, the Health and Safety Laboratory, and the University of Liverpool, conducted a project to assess whether engineering students, as future workplace designers, had a basic understanding of safety and health risk issues relevant to their specific course of professional study [7]. Students’ understanding of safety risk issues and key concepts were assessed and as a result of limited understanding new safety-related teaching materials were integrated into existing course content, in liaison with key stakeholders. An e-learning package was subsequently developed. More than half of the students exposed to new teaching materials improved their knowledge of safety-related concepts by more than 10% [7].

The work of Wachtler and Troein [8] potentially provides a framework for the evaluation of safety-related content in curricula. In their research on cultural competency, they reviewed learning outcomes, interviewed program directors and lecturers about course content, and conducted focus groups with students. The study reviewed curricula through three different perspectives: (1) the intended curriculum as designed by the academic; (2) the taught curriculum as delivered to students, and (3) the received curriculum as reflected in student experience.

With reference to the methodology of Wachtler and Troein [8], we undertook a previous pilot project, at the University of Adelaide, to assess safety content in undergraduate programs in the Schools of Nursing and Mechanical Engineering. These Schools were selected as dichotomous examples of the future workforce—designers and “end-users”—both of whom are critical to improvements in the field of workplace safety and injury prevention. Course handbooks were scanned for the inclusion of safety-related content and semi-structured interviews were conducted with academic program coordinators. While there was an absence of safety-related content in course handbooks, program coordinators considered that safety-related content was integrated in their programs. A Safety Content Assessment Tool (SCAT)—as a means to identify this “hidden” content—was then developed and subsequently piloted. Subsequent semi-structured interviews with the program coordinators indicated that the SCAT provided them with a means to identify existing safety content—or its absence—in their curriculum.

Due to the absence of published literature on the inclusion of safety content in university curricula, or a tool with which program or course content can be evaluated from a safety content perspective, the aims of the study reported here were twofold. First, to expand on our pilot project by exploring the views of final year undergraduate students, academic program coordinators and representatives of professional organizations and accrediting bodies with regard to existing safety-related curriculum content and the subsequent level of preparedness of graduates entering the workforce. Second, to validate the SCAT—which was developed during the previous pilot project—by conducting usability testing in three areas, namely undergraduate nursing, mechanical engineering, and education programs at all three major universities in South Australia (the University of Adelaide, University of South Australia, and Flinders University). The future “teachers” workforce (education) was included for its facilitation of safety-related skills, knowledge, and attitudes in young and/or new workers, who are over-represented in injury statistics [9].

## 2. Methods

A qualitative research approach was used to address the aims of the study within the framework described by Wachtler and Troein [8].

### 2.1. Interviews and Focus Groups

Semi-structured interviews were conducted with representatives of engineering, nursing, and education professional organizations, university academic coordinators (who also completed the SCAT), and final year undergraduate students. One focus group, comprising academic nursing staff at Flinders University, was conducted in order to accommodate their preference not to attend individual interviews. All interviews (and the focus group) were audio recorded and transcribed.

The interviews (and focus group) were based on semi-structured questions and were conducted by the same researcher (A.S.). The interviews were between 20–45 min in length. The focus group was of one-hour duration.

The questions for representatives of professional organizations included their understanding of safety, how they felt it was related to their profession, how prepared university graduates were for entry to the workforce, and opportunities and barriers for improvement in safety-related content in university curricula. Questions for academic coordinators included their own understanding and qualifications in workplace safety, its inclusion in the existing curriculum, the sources of information they were aware of, and regularly accessed, and student preparation for work placements. Questions for students included their understanding and the relevance of workplace safety, their experience of safety issues as an undergraduate how they were assessed, and how well prepared they felt, from a safety perspective, for their first student placement and after graduation.

### 2.2. Recruitment and Ethics

The process of accessing participants for interviews was initially via email/telephone contact. University academic coordinators also assisted in approaching specific students for interviews. Students were also accessed by asking participants if they knew of any interested peers who would be interested in being interviewed. Ethics approval was granted by the University of Adelaide (HS-2013-042).

### 2.3. Data Analysis

Audio recordings were transcribed prior to thematic analysis as described by Braun and Clarke [10]. This consisted of analyzing the transcripts and creating a list of themes based on participant statements. The transcriptions of each interview were read, re-read, and coded independently by two members of the research team. Open coding was conducted by identifying and labelling each idea or concept. Where labels coincided, this allowed for comparison between transcripts. These were then grouped together to develop themes that were compared between researchers. In order to maintain analytical coherency, the three groups (nursing, education, and mechanical engineering) were analyzed separately.

### 2.4. Safety Content Assessment Tool

Program coordinators from nursing, mechanical engineering and education from each of the universities were asked to apply the SCAT (Appendix A) to their existing curriculum. The responses were analyzed for each of the tool’s components. For the purposes of this study, a term more familiar to program coordinators—Occupational Health and Safety—was used in the tool instead of the more generic term of “safety”.

## 3. Results

Altogether, 20 interviews and one focus group were conducted. Three interviews were conducted with representatives of professional organizations—the Australian Nursing and Midwifery Federation, Australian Education Union, and Engineers Australia. Academic coordinators from the three participating universities—the University of Adelaide, Flinders University, and University of South Australia—were involved in five interviews and one focus group (nursing staff from Flinders University). The responses from the four-academic staff from the University of Adelaide who participated in the pilot study were also included.

In relation to students, five interviews were conducted with nursing students from the University of Adelaide and Flinders University (all females between 18 and 21 years of age). Four interviews were conducted with education students from the University of Adelaide, Flinders University, and University of South Australia (all females between 18 and 21 years of age). Three interviews were conducted with mechanical engineering students from the University of Adelaide, Flinders University, and University of South Australia (all males between 19 and 23 years of age).

### 3.1. Perspectives of Professional Organizations

#### 3.1.2. Safety in the Curriculum

All representatives agreed that workplace safety was an essential component in university curricula—one referred to it as a “necessary evil”. There was a consistent view that there was a general lack of awareness by students as to how safety is managed in the workplace. This was associated with discrepancies between the safety content in university curricula and actual workplace practices. They considered that universities are not adequately equipped to provide the optimal level of safety training which is needed in the workplace. However, they did acknowledge the limitations associated with university curricula.

#### 3.1.3. Safety Preparation for the Workforce

There were contrasting views on how well prepared new graduates were. While the engineering representative considered that engineering students were sufficiently prepared the education representative considered that new teachers were not. The nursing representative referred to the preparation of new graduates as a “mixed bag” due to differences in the curricula between universities.

All three bodies agreed on the need for improving the relevance of workplace safety content in existing curricula however they acknowledged the limitations to doing so:

I am not certain that it’s ever possible for any academic program to prepare a graduate that is absolutely work-ready without the employer ultimately having some responsibility at that point of the person commencing in work to do some top-up education and training in relation to workplace safety.

(Engineering representative 2)

### 3.2. Perspectives of Academic Coordinators

#### 3.2.1. Safety in the Curriculum

Academic coordinators considered that workplace safety content was embedded in all three curricula, both formally and informally. However, there were differences in opinion on the degree to which this knowledge was formally assessed.

Nursing coordinators agreed that workplace safety was embedded in the curriculum with various forms of assessment. For example, students were assessed at the completion of nursing intensive work placements on their clinical practice and their interaction with patients. Moreover, students were assessed on workplace safety aspects when working in simulated environments prior to work placements:

It’s embedded in the assessments that they do, particularly the practical assessments.

(Nursing coordinator 1)

Engineering coordinators were divided whether safety knowledge was formally assessed, with one view that it should be considered as common-sense:

… for me, occupational health and safety is common sense … if it’s really basic you assume other people around you have already considered it.

(Engineering coordinator 4)

Education coordinators agreed that workplace safety was not formally assessed in the curriculum. One commented that they were unsure as to why this was the case:

It’s not, and I guess I’ll be going away and having a little look at that to see why not.

(Education coordinator 2)

Engineering coordinators were more inclined to focus on the legalistic nature of workplace safety in the University curricula:

Engineering is certainly a discipline where there are potentially workplace hazards in the university environment, of course, and in industry as well, and that requires that we make sure that our students have an appreciation of health and safety issues when they leave the courses here.

(Engineering coordinator 2)

Nursing and education coordinators placed greater emphasis on the more social aspects of workplace safety and the need to monitor individual stress levels and mental health:

So when we’re talking about health, safety and wellbeing we’re talking about the wellbeing of the whole person not just the bits of the person—the physical self but also the mental self, the emotional self.

(Education coordinator 1)

#### 3.2.2. Safety Preparation for the Workforce

Coordinators from all disciplines considered that graduates were prepared, from a safety perspective, upon entering the workforce. However, their perceptions on the degree of preparedness differed:

No matter how much you educate them or try to get them to be alert to things... there are still people who will not recognize a situation as being something reportable….

(Nursing coordinator 2)

I don’t think we could ever teach a student so they can hit the ground running and be fully competent.

(Engineering coordinator 3)

### 3.3. Perspectives of Final Year Students

#### 3.3.1. Safety in the Curriculum

There was general agreement among students that workplace safety is embedded within the curriculum rather than being presented as a separate or “stand-alone” topic.

I think it was mingled throughout the entire three-year course.

(Nursing student 3)

Students across all three disciplines reported different levels of safety-related assessment in their curriculum. Engineering and education students reported less-formal assessment procedures, with some not recalling any form of assessment.

There was a pass and fail if you didn’t get a good enough score, whatever it was, and then you wouldn’t proceed. But it was pretty relaxed, I would say. It wasn’t so formal.

(Engineering student 4)

In contrast, nursing students reported both formative and summative competency assessment within their curricula:

We also have a lab where we re-enacted the clinical situation and we were watched and observed and did formal assessments which did contribute to our summative assessment.

(Nursing student 3)

#### 3.3.2. Safety Preparation for the Workforce

Education students raised concerns as to whether they had sufficient workplace safety knowledge when entering the teaching profession:

I guess a lot of that sort of preparation does get handled by the schools more so than the university because it is that natural workplace setting so I’m not sure how well this school will be preparing me for occupational health and safety issues.

(Education student 4)

Engineering and nursing students agreed that the university curriculum had prepared them for the workforce from a safety perspective:

I think we’ll be well prepared when we get into the workplace and then they’ll sort of run us through all that sort of stuff anyway so I think—well, I’m pretty confident that it should be okay.

(Engineering student 1)

#### 3.3.3. Suggestions for Change

Students consistently commented on the need for improved specificity and relevance in workplace safety content. Nursing students considered they did not learn enough in relation to patient handling during their undergraduate training and felt that the content should be more holistic in nature. For example, one participant suggested that more attention needed to be paid to the physiological and lifestyle effects associated with shift work such as poor eating habits, lack of exercise, and disrupted sleep patterns that may impact on musculoskeletal injury and the increased prevalence of obesity.

Similarly, education students suggested the presentation of more real case scenarios instead of the more general safety content which is currently delivered. They were particularly concerned about the management of workplace bullying and stress which had affected them during their undergraduate placements:

I ended up being so stressed at the placement that I ended up not eating until I went home—like hadn’t eaten breakfast, hadn’t eaten anything at the school all day and just was so stressed.

(Education student 4)

Engineering students also emphasized the importance of making safety-related curriculum content more relevant to their profession. They suggested the development of a safety-related learning laboratory on campus as a potentially significant educational experience.

### 3.4. Perspectives of Accrediting Bodies

Accrediting bodies may be seen as drivers of curriculum change in universities. Despite numerous attempts by telephone and written communication, access to representatives was not gained. Thus the views of accreditation bodies on workplace safety integration remain unexplored.

### 3.5. The Safety Content Assessment Tool

Academic coordinators from nursing, mechanical engineering and education from each of the universities were asked to apply the tool (Appendix A) to their existing curriculum. It was completed by nine academic coordinators. These were collated and the responses analyzed for the each of the tool’s components.

#### 3.5.1. The Intended Curriculum

While all course coordinators were affirmative in including workplace safety activities in their intended curriculum, the required actions for improvement differed between the professions. Nursing coordinators identified relatively few needed actions, while engineering coordinators focused on the need to ensure that technical aspects of safety were adequately covered. Education coordinators identified areas for potential improvement primarily related to preparedness for student placements.in the intended curriculum.

#### 3.5.2. The Taught Curriculum

Course coordinators generally considered that their taught curriculum (as delivered to students) was appropriate—mirroring the intended curriculum—while acknowledging that this would change with any alterations to the intended curriculum.

#### 3.5.3. The Received Curriculum

There were mixed responses by the course coordinators in relation to assessments which specifically include safety elements that were either intended or taught. Nursing and engineering curricula including more formal assessment components than education.

## 4. Discussion

Using a multi-stakeholder perspective, we sought to examine the integration of safety content in three key disciplines (nursing, engineering, and education), perceived effect on work-readiness and to validate a tool which could be used by academic coordinators to identify present, absent, and hidden aspects of the coursework curricula pertaining to workplace safety principles.

### 4.1. Safety in the Curriculum

While the importance of embedding workplace safety content in university curricula was emphasized by professional organizations, and academic coordinators considered this to be the case, both of these stakeholder groups acknowledged that its effectiveness was limited. Professional organizations attributed this to the inherent limitations associated with university curricula and the inability of universities to fully prepare their graduates for work entry. These limitations were largely mirrored in the responses of academic coordinators. While academic coordinators did consider workplace safety content to be embedded in the curricula, there were notable differences in the degree to which this was formally assessed. Nursing coordinators reported a variety of formal assessment procedures in simulated laboratories and real work environments while engineering coordinators reported a focus primarily on the legal aspects. However, workplace safety was described by one engineering coordinator as “common sense.” In contrast to these varying levels of assessment, teaching coordinators reported a general absence of formal assessment in their curriculum.

The varying approaches to assessment were reflected in the views of final year students. While students from each of the groups considered that workplace safety content was embedded in their programs the depth of this differed markedly. Nursing students commented on the variety of assessments, both formative and summative, they were required to complete. Engineering and education students, however, were generally unable to recall any formal assessment and where they did, described it as “relaxed.” The determination of competence in the absence of any formal assessment is anathema in university pedagogy, particularly where practical skills need to be demonstrated. While experiential learning is important, students traditionally pay particular attention to aspects of the curriculum on which they expect to be formally assessed. This can be likened to the inevitable student question, “Will this be in the exam”?

### 4.2. Safety Preparation for the Workforce

The varying levels of workplace safety content (and assessment) were reflected in the varying levels of students’ preparation for the workforce.

Perhaps unsurprisingly, academic coordinators from all three disciplines considered that their graduates were sufficiently prepared, from a safety perspective, to enter the workforce. However, they did acknowledge that the level of preparation may not be optimal.

The views of the professional organizations were more variable. The engineering discipline considered that new graduates (from a program that focused on the legalistic aspects of workplace safety) were sufficiently prepared for the workforce. This was not the view of the education discipline. Although nursing graduates had the most nuanced and comprehensive workplace safety content, the professional body viewed new graduates as a “mixed bag” from a safety perspective.

This pattern of responses was similar among final year students. Education students raised concerns regarding their level of workplace safety preparedness, while engineering and nursing students considered themselves to be adequately prepared.

### 4.3. The Safety Content Assessment Tool

In contrast to previous studies [3,7], our intent was to validate a generic tool which could be used by academic program coordinators to identify present, absent, and hidden aspects of coursework curricula pertaining to safety principles. By piecing together this safety “jigsaw puzzle” they could identify existing content and current gaps. This served to provide positive feedback where existing content was identified but also to highlight future areas for improvement and integration. Because hazards and risks are occupation-specific, we did not seek to develop a package of information to be integrated into course content but rather to assist program coordinators in the identification and modification of material. We considered that this approach would provide maximum flexibility for the integration of workplace safety concepts. The tool was designed as a quick “self-check” tool rather than an external audit or research tool. Consequently, it was purposely designed with “closed” questions to reduce any perceived administrative burden and to facilitate its ease of completion. However, even with a group of highly motivated coordinators, compliance with its completion was difficult with several of the coordinators requiring multiple reminders.

In common with Stacey et al. [7], we found that a project of this nature can only be undertaken with the close cooperation of academic staff with specific responsibilities, and the ability, to influence or modify existing course content. By default (demonstrated by their willingness to participate) those who participated in our study had a pre-existing awareness of the importance of workplace safety information. Reviewing course content across an entire multi-year program allowed them to consider how workplace safety information could be integrated and expanded over time as the students’ knowledge and profession-specific expertise increased. This progressive integration is supported by sound pedagogical principles, in accordance with Bloom’s taxonomy [11] by reinforcing important safety principles as student knowledge and experience increases. In the absence of these close cooperative links, it is likely that academics would be reluctant to review and modify course content without an external driver such as a formal course review or accreditation process.

The strength of this study is the multi-discipline and multi-stakeholder perspective. However, one must consider the implications of personal bias among academic coordinators undertaking a self-assessment of their own program. Studies of self enhancement bias, the tendency to describe oneself more positively than a normative criterion would predict, indicate that most individuals self-enhance and expect others to do so as well [12]. Self-assessment in education may differ from that of peers or mentors, or otherwise be less accurate when compared with actual performance [13]. Individuals may also be overconfident with newly (particularly rapidly) learned skills [13].

A further limitation of the toolkit is that it does not specifically address the needs of students from non-English speaking backgrounds (NESB) in the university setting. The impact of the formulation of the toolkit on students from NESB backgrounds in relation to comprehension and cultural differences must not be ignored. Future revisions of the toolkit could provide a focus on learning outcomes for these students.

The comments on the assessment tool made by the course coordinators were positive and the self-assessment was seen as a useful framework. However, it is, in its current form, a relatively static document. The tool would benefit from further input from end-users both in its format usability and presentation. The development of an engaging multi-media interface may increase its usability, compliance and adoption by academics, as noted by Stacey et al. [7] in their development of an e-learning package for undergraduate engineering students. Multimedia elements could include a brief video introduction about the importance and relevance of the review, along with testimonials and completed case studies. 

In order to be widely applicable the tool was developed as a generic resource. For internal use the tool would benefit by the inclusion of more specific information (i.e., internal or external resources) by university faculties/schools following adoption to tailor the tool to their educational goals.

In relation to location and monitoring, accessibility of the document by program coordinators is critical. The optimal location is likely to vary between universities. However, University Quality & Review Units and Health, Safety & Wellbeing Units may have the necessary infrastructure to host the document internally at a central location. Given their specialized focus on hazard management and risk assessment, it is imperative that the toolkit not be used as an audit tool by such central units, although be accessible and readily utilized by coordinators of varied disciplines within the university to evaluate their programs. It is vital that despite the currently heavily regulated and crowded curricula, that academics integrate workplace safety education into their coursework rather than adopt safety education as an ‘add-on’ to the curriculum.

Finally, accrediting bodies have the potential for significant influence in university curricula and their views are important. Further work should explore their perspectives.

## 5. Conclusions

In our study, there was no lack of desire for the integration of workplace safety content but rather a lack of awareness of where such content was already incorporated in multi-year programs, how it could be included if absent, and how it is perceived by graduating students. A Safety Content Assessment Tool can support the recognition of the integration of workplace safety content in curricula and enhance its inclusion so as to help achieve its social purpose.

## Appendix A—Safety Content Assessment Tool

**Element 1—The Intended Curriculum**

**Scope:** The relevance of occupational health and safety (OHS) for a particular course or program can be determined through examination of the program and course information, particularly learning outcomes.

**Program Information**	**Current Status**	**Action Required**	**Priority**	**Responsible**	**Date**	**Useful Resources**
Does the program include activities which may expose participants to OHS risks or hazards? If yes, have the courses containing these activities been identified?						
Does the program seek to produce graduates who will be involved in the design of infrastructure, products or services and/or management of work environments with health and safety implications? If yes, have the courses seeking to develop these skills been identified?						
**Course Handbooks**	**Current Status**	**Action Required**	**Priority**	**Responsible**	**Date**	**Useful Resources**
Do relevant Course Handbooks define potential OHS risks and hazards and their control mechanisms?						
Do relevant Course Handbooks identify appropriate responsibilities and accountabilities for staff and students (i.e., duty of care)?						
Are competencies in the management of OHS hazards included as assessable course components?						

**Element 2—The Taught Curriculum**

**Scope:** Examination of the taught curriculum offers insight into more practical aspects of OHS. The taught curriculum includes online content, lectures and lecture notes, practical work and field placements. Each of these elements should be examined to identify existing OHS content and opportunities to integrate additional information.

**Online Content—Learning Management System**	**Current Status**	**Action Required**	**Priority**	**Responsible**	**Date**	**Useful Resources**
Does online material include links to relevant Legislation, Regulations, Standards and Codes of Practice?						
**Lecture Content**	**Current Status**	**Action Required**	**Priority**	**Responsible**	**Date**	**Useful Resources**
Do lectures contain information related to relevant OHS hazards, risk assessments, obligations and responsibilities?						
**Lecture Notes**	**Current Status**	**Action Required**	**Priority**	**Responsible**	**Date**	**Useful Resources**
Do lecture notes or readings contain information related to relevant OHS hazards, risk assessments, obligations and responsibilities?						
**Practical Work/Field Placement**	**Current Status**	**Action Required**	**Priority**	**Responsible**	**Date**	**Useful Resources**
Is student competence in OHS (e.g., hazard and risk assessment) undertaken prior to any practical work or field placement?						

**Element 3—The Received Curriculum**

**Scope:** The received curriculum can be examined in terms of (1) students’ knowledge (i.e., formal assessment) and (2) students’ perceptions, e.g., feedback received from students in the form of Program/Course Evaluations that are now conducted for most courses. Of particular interest is students’ knowledge of rights and responsibilities, an aspect of OHS that can be applied to any workplace or situation.

**Course Assessment**	**Current Status**	**Action Required**	**Priority**	**Responsible**	**Date**	**Useful Resources**
Does course assessment include a formal OHS component, possibly as a sub-category of larger assessment criteria, where relevant?						
**Course/Program Evaluation**	**Current Status**	**Action Required**	**Priority**	**Responsible**	**Date**	**Useful Resources**
Is there scope in Course or Program evaluations for students to provide feedback or comment on relevant OHS course content?						
